# DUckCov: a Dynamic Undocking‐Based Virtual Screening Protocol for Covalent Binders

**DOI:** 10.1002/cmdc.201900078

**Published:** 2019-03-08

**Authors:** Moira Rachman, Andrea Scarpino, Dávid Bajusz, Gyula Pálfy, István Vida, András Perczel, Xavier Barril, György M. Keserű

**Affiliations:** ^1^ Facultat de Farmàcia and Institut de Biomedicina Universitat de Barcelona Av. Joan XXIII 27–31 08028 Barcelona Spain; ^2^ Medicinal Chemistry Research Group Research Centre for Natural Sciences Hungarian Academy of Sciences Magyar tudósok körútja 2 1117 Budapest Hungary; ^3^ Laboratory of Structural Chemistry and Biology & MTA-ELTE Protein Modelling Research Group Eötvös Loránd University Pázmány Péter sétány 1/A 1117 Budapest Hungary; ^4^ Catalan Institution for Research and Advanced Studies (ICREA) Passeig Lluís Companys 23 08010 Barcelona Spain

**Keywords:** covalent docking, dynamic undocking, targeted covalent inhibitors, virtual screening

## Abstract

Thanks to recent guidelines, the design of safe and effective covalent drugs has gained significant interest. Other than targeting non‐conserved nucleophilic residues, optimizing the noncovalent binding framework is important to improve potency and selectivity of covalent binders toward the desired target. Significant efforts have been made in extending the computational toolkits to include a covalent mechanism of protein targeting, like in the development of covalent docking methods for binding mode prediction. To highlight the value of the noncovalent complex in the covalent binding process, here we describe a new protocol using tethered and constrained docking in combination with Dynamic Undocking (DUck) as a tool to privilege strong protein binders for the identification of novel covalent inhibitors. At the end of the protocol, dedicated covalent docking methods were used to rank and select the virtual hits based on the predicted binding mode. By validating the method on JAK3 and KRas, we demonstrate how this fast iterative protocol can be applied to explore a wide chemical space and identify potent targeted covalent inhibitors.

## Introduction

Despite the activity of a large number drugs approved by the US Food and Drug Administration (FDA) that depend on a covalent mode of action,^1^ classical drug discovery screening cascades typically eliminate electrophilic compounds, mainly due to the toxicity risks associated with their mechanism. Indeed, a majority of these drugs was discovered by serendipity in biological assays, and their mechanism was elucidated later on, typically after approval. The reluctance to use reactive ligands, and more specifically promiscuous “suicide inhibitors”, is related to increased risks of carcinogenicity, hepatotoxicity, and potential idiosyncratic effects caused by protein haptenization.^2^, ^3^

More recently, the reputation of covalent binders has changed thanks to the guidelines introduced for the rational design of targeted covalent inhibitors (TCIs). According to these guidelines, the ligand's selectivity toward its protein target is still to be achieved by optimizing the noncovalent interactions (hydrogen bonding, van der Waals, electrostatic, etc.) at the binding site, as in the case of traditional approaches. Furthermore, increased specificity can be obtained by targeting a poorly conserved reactive residue within the protein family.^3^ To this effect, the development of methods to identify poorly conserved reactive residues have aided the acceleration of TCI design. For example, activity‐based protein profiling techniques (ABPP, isoTOP‐ABPP^4^, ^5^) can be used to both investigate the activity at the proteomic level and quantify the intrinsic reactivity of functional cysteines. Also, Liu and colleagues have coined the term “kinase cysteinome” to refer to the collection of targetable cysteine residues in the human kinome^6^ and published a computational methodology to identify such cysteines.^7^

Ligands that bind through a covalent mechanism are not subject to classical equilibrium kinetics, as their residence time in the binding pocket can last up to days. As a consequence, the potency of these drugs is capable of surpassing the theoretical limits of potency/ligand efficiency.^2^ Another advantage is the prolonged duration of action, which can persist even when the ligand has already been cleared from the body. This can be beneficial for alleviating the drug burden of a patient due to less frequent drug dosing (depending on the turnover rate of the protein) and therefore a possibly lower risk of idiosyncratic toxicity, which has been linked to daily drug dosage.^8^

In addition, the TCI approach has proven to be a valuable tool in targeting protein binding sites, which were previously considered as undruggable, as well as to combat drug resistance by targeting poorly conserved non‐catalytic residues. Overall, all of these aspects have contributed to a resurgence of covalent drug discovery programs, which has already led to an increase of clinical candidates acting via a covalent mechanism.^9^

In general, a covalent binder first requires the formation of an initial noncovalent complex with its target, followed by the chemical reaction between the ligand's electrophilic warhead and the nucleophilic residue. As such, the most straightforward covalent drug design approach is based on the modification of a known noncovalent binder to introduce an electrophilic warhead. This could indeed allow to reach and covalently modify the targeted nucleophile on the protein by maintaining the overall binding mode in the rest of the pocket. Additionally, an important strategy is to fine‐tune the warhead reactivity based on the target nucleophilicity in order to limit possible side effects arising from off‐target modifications.^9^, ^10^, ^11^

From a computational perspective, once an appropriate nucleophile and warhead are identified, a structure‐based approach can be used to screen or optimize ligands to fit the binding site, while also being able to place the warhead in the vicinity of the targeted residue to form the covalent bond. Several covalent docking methods have recently been developed to model the structural changes occurring when covalent ligands bind to their target. However, other than the inherent limitations of traditional docking methods (i.e., scoring, protein flexibility, solvation, and nonclassical effects),^12^ these tools also have to address additional challenges in the simulation. Predicting the optimal geometry of the reacting groups upon covalent bond formation is of key importance for accurate simulations. Furthermore, covalent docking programs face the inability to evaluate the energy of bond formation, which would require QM‐based simulations of the reaction. Depending on the method of choice, modeling all the different and key aspects characterizing the binding of covalent ligands is often reflected in higher computational costs than for traditional noncovalent docking.

Among the first developed tools are GOLD^13^ and AutoDock:^14^ the former enforces the covalent reaction through the definition of a link atom in both the ligand and receptor before initiating the genetic algorithm search, while the latter offers the opportunity to choose between the *two‐point attractor* approach and the better performing *flexible side‐chain* method, in which the ligand is sampled as part of the protein. In addition to a lack of the energetic contribution of covalent binding, the manual definition of the atoms involved in the reaction hinders the applicability of covalent docking programs to large libraries. A recent approach taken by CovalentDock^15^ automatically detects reactive atoms for linking and rewards the energy contribution of the binding event as an additional MM‐based term. The authors retrospectively validated their method on 76 covalently bound ligands in the Protein Data Bank (PDB), for which CovalentDock showed better performance than GOLD and AutoDock. However, CovalentDock is limited in reaction types (only Michael addition and β‐lactam opening are supported) and does not account for the flexibility of the reacted residue. Furthermore, the cloud web server developed for its usage appears to no longer be available (access attempted on October 16, 2018). More recently, other web‐based servers such as DOCKovalent,^16^ or proprietary software such as ICM‐Pro,^17^ FITTED,^18^ and DOCKTITE^19^ (an SVL‐based workflow for the modeling software MOE^20^) enabled covalent docking‐based virtual screening applications by using predefined and customizable reactions to identify reacting groups.

Schrödinger's CovDock^21^ takes it one step further and mimics the full binding process of covalent ligands (as opposed to only taking into account the covalently attached ligand–protein complex). With this, CovDock highlights the importance of the noncovalent interactions formed prior to covalent binding. The multistep algorithm provides two alternative solutions by means of a “pose prediction” module and a virtual screening module (CovDock‐VS). The former includes an extensive protocol for the prediction of the covalently bound pose, namely: I) ligand conformation generation; II) positioning the pre‐reaction form of the ligand warhead close to the receptor reactive residue (mutated to Ala) using a constrained docking; III) resetting the mutation to the original residue, sampling its rotameric states, and generating the covalent attachment; IV) clustering and minimization of the poses (including the reacted residue); and V) scoring by means of the Prime energy model. An additional affinity score, which averages GlideScore on both the pre‐ and post‐reaction forms of the ligand, is provided to compare different compounds equipped with the same or similar reactive warheads. While it shows good binding mode prediction accuracy, this protocol takes roughly 1–2 CPU hours per ligand, so it is not suited for high‐throughput screenings. Toledo Warshaviak and colleagues addressed this issue by developing CovDock‐VS,^22^ which I) skips the ConfGen step, II) limits the number of resulting pose clusters to three, III) excludes minimization by Prime, and IV) scores and ranks protein–ligand complexes based only on the initial GlideScore. Ultimately, this led to significantly improved speeds (≈15 minutes per structure on a single CPU according to the info on CovDock's latest release) over the pose prediction module, but also yielded less accurate binding mode predictions, unless known interaction patterns were incorporated.

**Figure 1:**
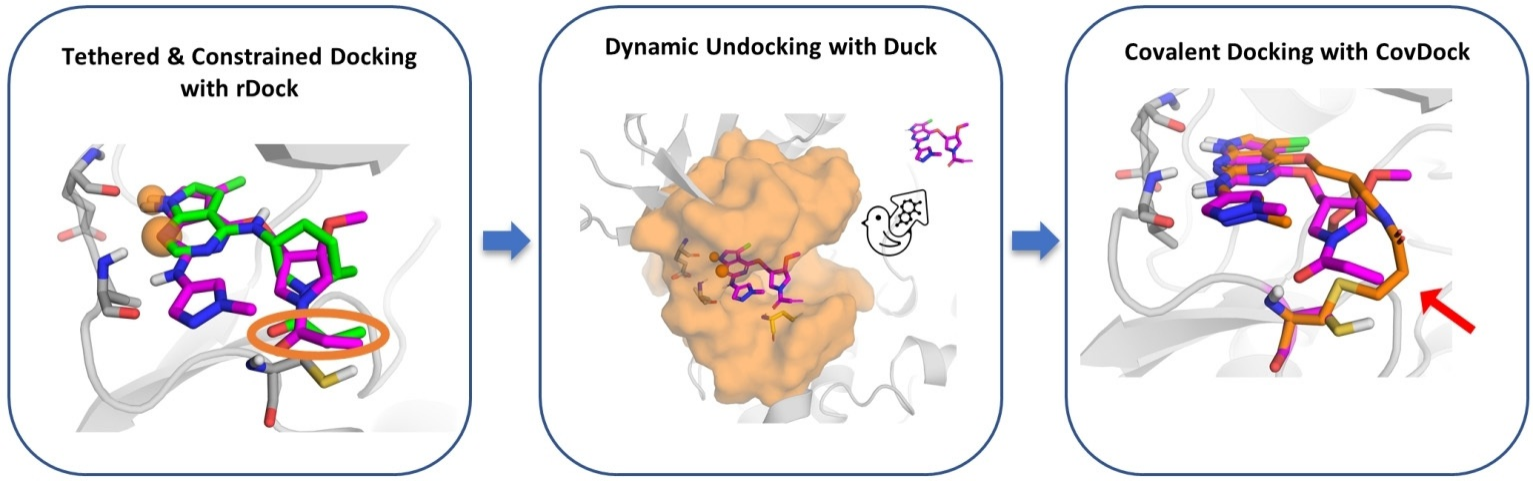
Starting from a library of covalent ligands, the general workflow is as follows: 1) docking with rDock with pharmacophoric constraints (orange spheres) and positional restraints for the warhead (encircled by orange ellipse), 2) dynamic undocking to test the strength of the H‐bond interaction that was enforced during docking, and 3) covalent docking of ligands that display the best noncovalent interactions to account for warhead flexibility (red arrow).

In general, the performance gap in terms of binding mode prediction among the different covalent docking programs was shown to vary significantly depending on various factors (i.e., protein target, accessibility of the nucleophilic residue, amount of noncovalent interactions occurring in the complex).^23^ On the other hand, the speed of the simulation remains one of the main bottlenecks that can drastically affect the size and diversity of the covalent libraries used for screening applications. To this end, herein we present DUckCov, a time‐efficient multistep VS protocol for the identification of novel covalent binders. It was devised to emphasize the role of the interactions mediating the initial noncovalent complex, whose optimization can, therefore, result in both an increase of the selectivity for the target and in an opportunity to decrease the reactivity of the electrophile. As depicted in Figure 1, rDock^24^ is first used to constrain the reactive warhead close to the targeted residue. During docking, pharmacophoric restraints are applied to known H‐bond interaction points, if any. Dynamic Undocking (DUck)^25^ is then used to assess the strength of these H‐bonds. DUck evaluates structural stability, rather than thermodynamic stability, and has been shown to be orthogonal to methods that attempt to estimate the binding energy. H‐bonds are suggested to be the main determinants of structural stability based on their sharp distance and angular dependencies, and their role in structure‐kinetic relationships.^25^, ^26^ Finally, CovDock is used to evaluate the binding mode of those ligands that optimally bind through noncovalent interactions, and to check if the same interaction pattern is maintained in the predicted covalent docking pose.

The protocol was prospectively validated in two case studies: a target with highly conserved noncovalent interactions (JAK3) and another one where the noncovalent interactions are not conserved across known inhibitors (KRas^G12C^).

## Results and Discussion

### Case study 1: JAK3

JAK3 is one of the four Janus kinases (a subfamily of tyrosine kinases), the only of which is primarily restricted to leukocytes. Its functional modulation has been associated with a phenotype of severe combined immunodeficiency.^27^ Although significant effort has been put into the discovery of JAK inhibitors, the search for JAK‐specific ligands is still on‐going. JAK3 specificity over other family members can be achieved by targeting C909 with ligands that are able to covalently bind this residue. H‐bond interactions are known to play a prominent role in building up affinity toward kinase targets. Because the majority of kinase inhibitors bind to the highly conserved hinge motif, DUckCov application on JAK3 was focused on the identification of covalent ligands displaying strong interactions at this region.

**Figure 2:**
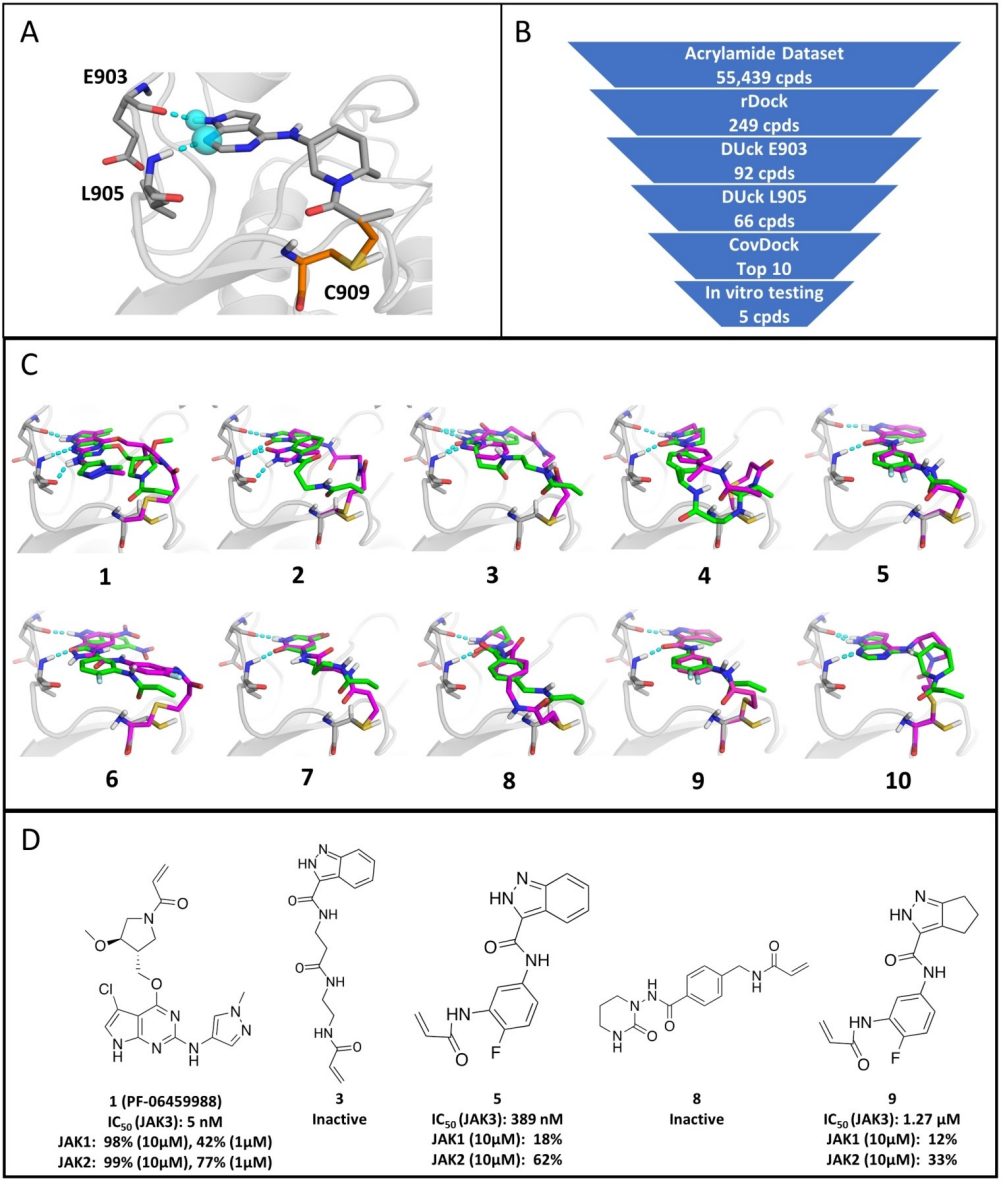
A) Pre‐reaction reference ligand in the structure 5TOZ in grey, and covalent attachment in orange in the post‐reaction form, with defined features as cyan spheres, and interactions in cyan dashes. B) DUckCov protocol for JAK3. C) Top‐ten compounds, ranked by CovDock affinity scores (green: rDock binding modes, magenta: CovDock binding modes; interactions with the hinge residues E903 and L905 are displayed in cyan). D) Experimentally tested compounds for JAK3; the three confirmed compounds correspond to a hit rate of 60 %.

The DUckCov workflow for JAK3 is described in Figure 2 B. Based on the selected JAK3 structure, tethered and constrained docking filtered the acrylamide dataset from roughly 50 000 compounds to 249 compounds that satisfied the H‐bond interactions with E903 (backbone C=O) and L905 (backbone NH) at the hinge region (depicted in cyan in Figure 2 A), while conserving the acrylamide group close to the reactive C909 as observed in the prepared reference ligand (depicted in grey in Figure 2 A). Next, DUck was performed, using the H‐bond established with E903‐O as the simulation coordinate, leading to 92 remaining hits. From those, a second round of DUck on the H‐bond formed with L905‐NH resulted in 66 compounds. In both cases, a W_QB_ (work necessary to pull the ligand from 2.5 to 5.0 Å relative to the defined H‐bond interaction) threshold of 6 kcal mol^−1^ was maintained. The consensus of both interactions was used to filter the rDock docking poses using DUck, as both these interactions are made by the reference ligand (with W_QB_=13 kcal mol^−1^ and 11 kcal mol^−1^, respectively). Then, in order to get both a quantitative ranking and more accurate binding mode predictions, CovDock in the “Pose prediction” module was used for the covalent docking simulations on the 66 DUck hits. Finally, we have selected the top 10 ligands according to their CovDock affinity scores. Out of these, five compounds (**1**, **3**, **5**, **8** and **9**) were available for immediate purchase (Figure 2 C–D): they were experimentally validated in an enzyme‐based activity assay (see Methods section). For the rest of the top 10 ligands, see Supporting Information Figure S6.

Compound **1,** the top‐ranked ligand, has been originally reported as a potent double mutant EGFR^L858R/T790M^ inhibitor (PF‐06459988, IC_50_=7 nm),^28^ and based on the DUckCov prediction and subsequent in vitro testing, we found that the compound inhibits JAK3 with similar potency (5 nm). Interestingly, JAK3 activity of this compound was not reported previously; however, profiling against 54 human kinases at 1 μm revealed its moderate JAK1 and JAK2 inhibitory activity.^28^ Compounds **5**, **6**, **8** and **9** are characterized by a higher rigidity of the linker between the hinge binding region and the warhead. Compound **5** displayed an IC_50_ value of 389 nm on JAK3, and 18 % and 62 % inhibition on JAK1 and JAK2 at 10 μm, respectively, suggesting a covalent bond driven improvement of the inhibition due to the presence of the JAK3 unique reactive cysteine. Compound **9**, a partially saturated analogue of **5**, was found to be less potent (1.27 μm IC_50_), in line with the observed vast majority of aromatic hinge binding moieties known in the literature. In the same line, compound **8** showed no activity in the biochemical assay, further highlighting the preference for planar hinge‐binding cores. Finally, compound **3** was also experimentally tested (as an analogue of **5** with a flexible linker), but has shown no activity.

In Figure 2 C the poses generated by rDock and CovDock for the top 10 ligands are shown in green and magenta, respectively. In nine out of 10 cases the interactions used for pulling with DUck were reproduced in the best scoring pose generated by CovDock. (RMSD values, CovDock affinity, rDock scores, and DUck W_QB_ values, along with the ZINC codes of the compounds are given in Supporting Information Table S7). Compounds **1**, **4**, **5**, **7**, **8**, **9** and **10** retain the same orientation of the hinge binding region, while compounds **2** and **3** contain the hinge binding scaffold in a flipped orientation, due to the flexibility of the linker. However, compound **3** maintained both interactions at the hinge, while compound **2** showed a dual interaction with L905. In addition to the higher deviations (RMSD) between the binding modes predicted by rDock and CovDock, the linker flexibility of compounds **1**–**4** results in higher strain energies as well (relative to minimum energies of the free ligands, see Supporting Information Table S8). However, this does not necessarily prevent the compounds to be potent inhibitors of JAK3 (as exemplified by compound **1**), in accordance with the general notion that the linker rarely has a profound effect on activity. Compound **6** is the only case where neither an interaction with E903, nor L905 is observed. (The ten lowest ranked ligands are included as counter‐examples in Supporting Information Figure S9, most of them lacking any kind of interaction with the hinge).

The 60 % hit rate observed with DUckCov against JAK3 demonstrates the importance of noncovalent interactions established by covalent ligands. However, these findings also clearly show the need for dedicated covalent docking programs sampling multiple rotameric states of the reactive residue. This would increase the chance to identify the most optimal geometry of the covalent attachment, which could consequently reflect in a rearrangement of the overall binding mode that would be generated by tethered docking.

It is also important to note that running the DUckCov workflow took a total 1200 CPU/GPU hours, while running CovDock Virtual screening on the whole dataset would have taken about 13 750 CPU hours (15 minutes per ligand according to the software manual). The roughly 11‐fold speedup can be mostly attributed to the quick tethered docking step, leaving only a fraction of the ligands to be evaluated by the more expensive Dynamic Undocking. If we account for parallelization as well, running this specific workflow in parallel on 24 GPUs of the Barcelona Supercomputing Center has required a total 50 hours of runtime, while our license token limit would have allowed us to run CovDock Virtual screening on three parallel threads (three ligands), resulting in about 4600 hours of total runtime, translating to a roughly 92‐fold decrease in speed compared to the DUckCov workflow. The reported speedups can be considered typical for academic groups (based on the accessible resources), but in a more general sense, CPU/GPU time is more accessible (cheaper) than state‐of‐the‐art software licenses (such as Schrödinger) for industrial researchers as well.

### Case study 2: KRas^G12C^

To challenge the method's applicability domain, it was also applied to another oncological target, the catalytic domain of KRas^G12C^. For KRas^G12C^, even the best irreversible binders show low potency if their covalent warhead is removed.^29^ KRas is a small G protein, which is rendered constitutively active by the G12C mutation, leading to abnormal cell growth. The mutation has been shown to be implicated in 40 % of KRas‐driven lung cancers.^30^ Known covalent ligands bind to a highly flexible allosteric pocket, which traps KRas^G12C^ in the inactive GDP‐bound state (thereby confirming its druggability).^31^ Additionally, covalent ligands can specifically target the mutated KRas^G12C^, sparing the wildtype protein and offering the opportunity for oncogene‐specific inhibition.^30^

In Table 1, the various H‐bond interactions are displayed for 10 KRas^G12C^ structures containing a covalently bound acrylamide ligand, as well as the W_QB_ values obtained for each interaction on the reference ligand. For the remaining two of the 12 selected structures (PDB IDs 4M21 and 6ARK), no H‐bonds could be reliably identified. An interaction was used in DUckCov if the work required to break the H‐bond was higher than 6 kcal mol^−1^. Thus, structures 5F2E (pulling from atoms R68‐NH2, E63‐O and D69‐OD1), 5V9O (pulling from atoms K16‐NZ, E63‐OE2 and D69‐OD1), and 5V6S (pulling from atom D69‐OD1) were used to validate the DUckCov protocol on KRas^G12C^.

**Table 1:** Interaction patterns for the selected KRas^G12C^ structures.^[a]^

PDB ID	LIG	Chain	R68 NH2 [Don]	K16 NZ [Don]	E63 O [Acc]	E63 OE2 [Acc]	D69 OD1 [Acc]	H95 NE2 [Don]
4M22	22C	B		6.5				
5F2E	5UT	A	10	X	10		13	
5V6S	8YD	A		X			9.2	X
5V71	8ZG	A		X				X
5V9L	91D	A		X				X
5V9O	91G	A		10		12	21	
5V9U	91S	A						X
5YXZ	94C	A		X				X
5YY1	94F	A		X				X
6B0V	C8G	A	X	X

[a] X indicates that the calculated DUck W_QB_ value was <6 kcal mol^−1^, otherwise the value corresponds to the work necessary to break the H‐bond during the DUck simulation in kcal mol^−1^.

**Figure 3:**
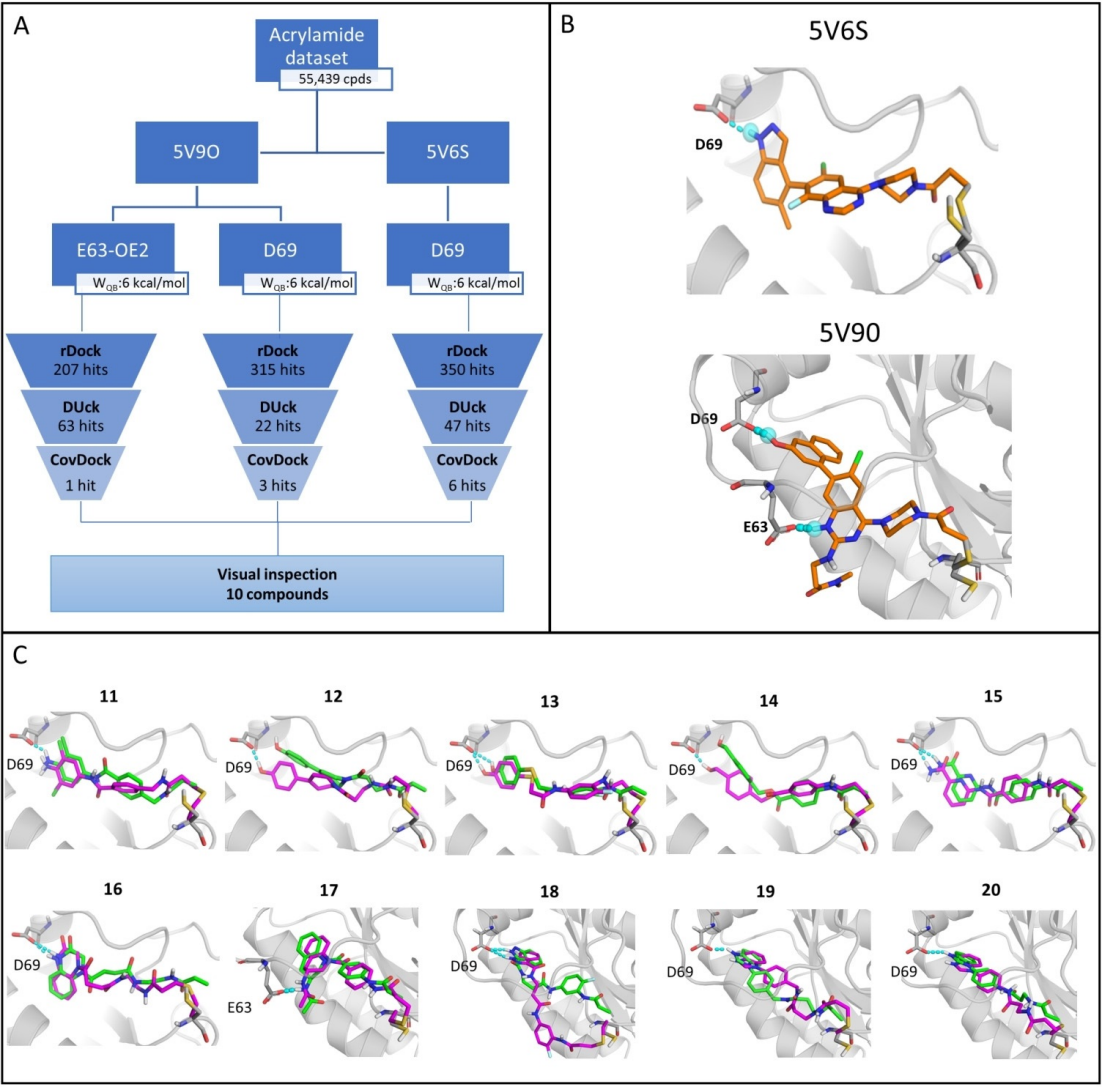
A) DUckCov workflow for KRas^G12C^ for the structures and interactions that eventually led to virtual hits. B) Pre‐reaction reference ligands in 5V6S and 5V9O in orange (warhead used for tethering), with covalent attachment in grey, and pharmacophoric features for docking/interaction for DUck in cyan. C) Compounds resulting from DUckCov workflow against 5V6S (**11**–**16**) and 5V9O (**17**–**20**). In the latter case, compound **17** was retrieved considering the feature/interaction with E63, while compounds **18**–**20** were retrieved considering the feature/interaction with D69. rDock poses and CovDock poses are shown in green and magenta, respectively.

In Figure 3 A, the workflow is summarized for the two structures and three interactions that led to virtual hits, namely 5V9O, E63‐OE2 and D69‐OD1, and 5V6S, D69‐OD1. Finally, 63, 22 and 47 hits were generated by DUckCov from these interaction points, respectively. Based on the results of JAK3, the compounds that were selected for experimental testing were ensured to have maintained the inspected H‐bond and/or displayed similar binding modes according to rDock and CovDock. The RMSD between rDock and CovDock poses and diversity of the hits were also used to support the final selection (Figure 3 C).

In Figure 3 C, the compounds retrieved using the stepwise workflow are shown. Compounds **11**, **13**, **15** and **16**, maintain the defined interaction with D69 of the KRas structure 5V6S, in both rDock and CovDock poses. It should be noted that the pharmacophoric restriction in rDock has a tolerance of 1 Å relative to the reference coordinates. As a result, in some cases the input geometry for DUck does not form a hydrogen bond. Yet, the initial step in the DUck protocol involves a minimization that can repair the H‐bond. For this reason, as shown in compounds **12** and **14**, some interactions present high W_QB_ values even though they were not recapitulated by rDock. These interactions are also present in the CovDock pose.

**Figure 4:**
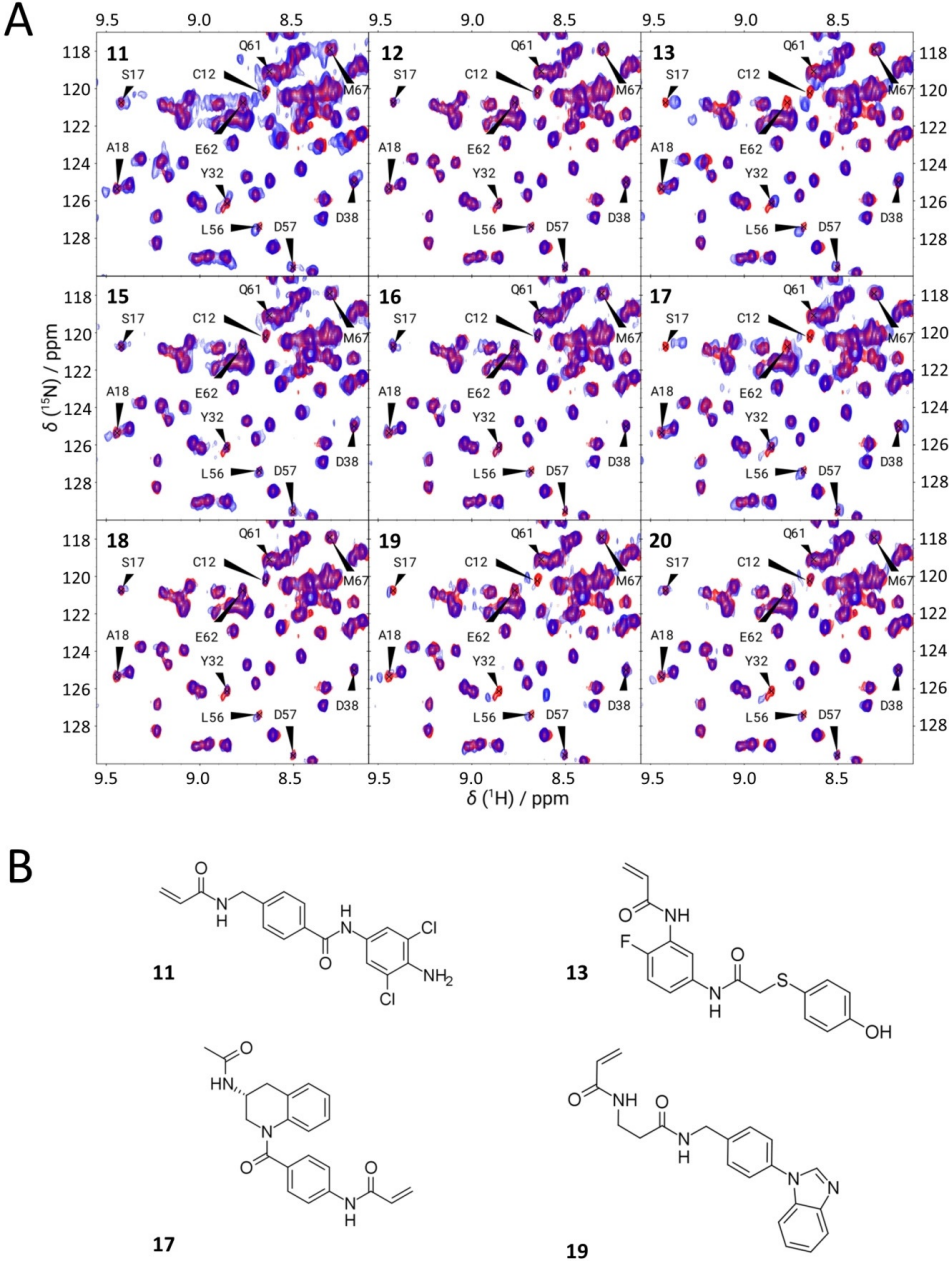
A) ^1^H,^15^N‐HSQC spectra of the tested nine molecules and KRas^G12C^, showing the spectral region of C12 and other binding site residues (blue), overlaid on the reference spectrum of the free protein (red), after incubation. For compounds **11**, **13**, **17** and **19**, most of the highlighted residues from the P‐loop (C12, S17, A18), Switch I (Y32, D38) and Switch II (L56, D57, Q61, E62, M67) regions are significantly perturbed, while almost no changes are detected for **12**, **16** and **18**. Small (inconclusive) changes are detected for compounds **15** and **20**. B) 2D structures of the experimentally confirmed hit compounds against KRas^G12C^.

The overall conservation of the binding modes in all ten compounds is reasonably good, in accordance with the RMSD values between the rDock and CovDock poses. RMSD values, CovDock affinity and rDock SCORE.INTER (interaction score energy) scores, as well as W_QB_ values, for the final ten compounds are reported in Supporting Information Table S10, along with their ZINC codes. These compounds were purchased and tested in HSQC NMR measurements (except for compound **14** that was not available for immediate purchase at the time of this study). The 2D structures are included in Figure 4 B for the confirmed hits, and Supporting Information Figure S11 for the rest. Strain energies of the resulting binding modes are reported in Supporting Information Table S12.

The site specific binding of compounds **11**, **13**, **17** and **19** was confirmed by ^1^H,^15^N‐HSQC (2D NMR) measurements. After the appropriate incubation time, changes in the HSQC spectrum were detected based on chemical shift perturbation confirming the binding of the mentioned small molecules (see Figure 4 A, and Supporting Information Figure S13 for the full spectra). The perturbed chemical shifts are located mostly in the well‐known Ras functional regions: the P‐loop (G10‐S17), Switch I (D30‐D38), and Switch II (L56‐G77). Overall, the findings suggest that the compounds can bind to KRas^G12C^ covalently at the C12 residue, and are located in the allosteric binding pocket of KRas, similarly to known inhibitors such as ARS‐853^31^ and ARS‐1620.^32^ It is worth to note that from the three protein structure/H‐bond combinations that were applied for the DUckCov workflow (Figure 3 A), all of them have produced at least one confirmed hit compound.

The 44 % hit rate retrieved for KRas using the DUckCov protocol is exceptional, considering the lower druggability of this target. Moreover, the correlation between affinity and activity remains elusive.^33^ Considering this, the protocol was successful in identifying four novel KRas covalent binders in an efficient manner by first focusing on the noncovalent interactions, even though these are known to be non‐conserved. Furthermore, a dedicated covalent docking program is imperative for the evaluation of possible rearrangements of the overall binding mode generated by rDock. By sampling multiple rotameric states of the reactive residue, forming the covalent bond between the reactive atoms and performing structural optimization of the covalently attached ligand, the binding mode prediction module in CovDock could increase the chance to find an optimal geometry for the ligands. Additionally, a comparison with Schrödinger's CovDock Virtual screening module clearly highlights the advantage of the DUckCov workflow, as three out of the four confirmed hits were not included in the top ten virtual hits by CovDock (Supporting Information Table S14).

## Conclusions

DUckCov is presented here as a novel protocol for the identification of covalent binders that models every stage of the multistep binding mechanism of covalent ligands in an efficient hierarchical manner. In this protocol, only molecules that can form a stable and productive pre‐reactive state are evaluated before assessing the post‐reactive state, thereby allowing to explore large chemical spaces. Dynamic Undocking (DUck) is the main feature of the workflow, as it is used to analyze the pre‐reactive state of the ligands by evaluating the strength of H‐bond interactions driving the formation of the initial noncovalent protein–ligand complex. Furthermore, DUck calculations are performed on focused protein chunks, thus enabling fast simulations by decreasing the size of the system. Therefore, DUckCov relies on DUck as a stringent and efficient filter for the selection of molecules to be subjected to the following steps. Next, the post‐reactive state is analyzed by performing covalent docking with CovDock in the most accurate pose prediction module. This step is used to generate bound conformations, thus allowing to compare binding modes in the pre‐ and post‐reaction states, and to assess if the key H‐bonds are maintained when the ligand is covalently bound to the targeted nucleophilic residue.

Our protocol was successfully validated in two case studies. For JAK3, we reported a hit rate of 60 % (three actives out of five molecules tested), identifying two novel, low micromolar and high nanomolar ligands, as well as a low nanomolar inhibitor, originally developed for another kinase target (EGFR). For the more challenging KRas^G12C^ protein target, four novel covalent ligands were experimentally confirmed out of nine tested. Due to the highly flexible nature of the KRas^G12C^ allosteric binding pocket, the resulting 44 % hit rate can be considered exceptionally good. The two case studies display the broad applicability of DUckCov, in identifying novel chemical matter for structurally better characterized, as well as more challenging targets. It is also important to highlight that, depending on the available resources, the presented workflow can provide a roughly ten‐ to hundred‐fold speedup, as compared to a commercially available virtual screening tool for covalent binders (Schrödinger CovDock).

## Experimental Section

**Target structure selection**: For JAK3, the PDB structure 5TOZ (chain A) was used as the template, using the co‐crystallized inhibitor PF‐06651600 as the reference ligand. At the moment of selection, eight structures were available containing covalently bound ligands having a terminal acrylamide as warhead (4QPS, 4V0G, 4Z16, 5TOZ, 5TTS, 5TTU, and 5TTV). Alignment and superposition of these structures in MOE^20^ led to an average RMSD of 0.78 Å. Given the structural conservation of the JAK3 kinase, the choice for structure 5TOZ was based on its co‐crystallized ligand having the best inhibitory potency (0.4 nm).^34^

For the second case study, a structure ensemble approach was used, due to the pronounced flexibility of the KRas allosteric binding site. At the end of May 2018, 23 KRas structures containing covalently bound ligands had been deposited in the PDB. The majority of unique ligands formed a covalent bond via Michael addition (20/23). Of these, 12 were acrylamide‐based covalent ligands, while eight contained a vinylsulfonamide moiety as the electrophile. As for the remaining three complexes, one was formed via a ring opening reaction (5V6V) and the other two were formed through disulfide formation (4LUC, 4LV6). We limited the set to the 12 acrylamide‐based complexes due to the limited commercial availability of screening compounds able to react via ring opening or disulfide formation, and the narrower chemical space of available vinylsulfonamides (roughly 2000 purchasable compounds in ZINC, versus 50 000 purchasable acrylamides^16^). The average RMSD of these 12 structures in the flexible switch II loop was 2.41 Å (Supporting Information Table S1). From these 12, those structures in which at least one H‐bond with the co‐crystallized ligand was stronger than 6 kcal mol^−1^ (as evaluated by DUck) were selected for the ensemble approach in DUckCov.

**Protein structure preparation (in silico)**: All of the selected PDB structures were prepared in MOE as follows: I) the structure was corrected (termini were capped, gaps were capped or a homology of the sequence of a similar structure was built, alternate conformations were chosen if more than one was present, correct tautomeric states for the residues were assigned); II) the structure (including the covalently bound ligand) was protonated at pH 7; III) the covalent bond between the residue and the ligand was manually broken; IV) the ligand warhead in its pre‐reaction form was built with MOE builder by making the acrylamide's Cα‐Cβ bond double, and finally; V) the cysteine was rebuilt, then minimized in the presence of the pre‐reaction ligand.

The structures for the CovDock simulations were prepared with the Protein Preparation Wizard provided in the Schrödinger Suite,^35^, ^36^ in order to further refine the protein's H‐bond network and to perform a restrained minimization of hydrogen atoms. The receptor grid box required for docking calculations was centered on the corresponding co‐crystallized ligand.

**Datasets for VS**: As one of the main features of the protocol is its efficiency, it is most beneficial when a large collection of electrophilic ligands is available. In general, if the protein has already been targeted by covalent inhibitors, the library can be compiled by collecting commercially available ligands and/or by enumerating synthetically accessible compounds bearing the same warhead type as the crystallized inhibitor. For JAK3, the vast majority of known covalent inhibitors bind through an acrylamide warhead, while for KRas^G12C^, most of the known potent covalent inhibitors bind through either an acrylamide or a vinylsulfonamide warhead. Further on, due to the limited commercial availability of screening compounds containing other types of warheads, we used the acrylamide dataset (roughly 50 000 compounds) collected by London et al. (for testing their recently published covalent docking program, DOCKovalent), to validate our protocol.^16^ Prior to docking simulations, LigPrep by Schrödinger was used to prepare 3D conformations from SMILES codes and to generate tautomeric and ionization states at pH 6–8 while retaining specified chiralities.^35^, ^36^

**General workflow description**: An overview of the protocol is shown in Figure 1. The collected library (here: ZINC acrylamide collection) is first docked with rDock against the target of interest, while simultaneously tethering the covalent warhead to its reference coordinates and using pharmacophoric constraints to enforce the main noncovalent interaction. Because the protein structure is derived from a crystallized covalent complex, a distance cutoff is set to avoid large deviations of the electrophilic warhead from the position defined in the reference ligand. H‐bond pharmacophoric constraints are applied in the docking simulation to keep only those ligands that can establish the H‐bond interactions defined as important for binding. DUck is then used to evaluate the strength of the H‐bond. For DUck, only H‐bonds are assessed, as they are known to be key contributors to affinity in many targets.^37^, ^38^ In a DUck simulation, the ligands are pulled from 2.5 to 5.0 Å relative to the defined H‐bond interaction point in the protein, during a user‐defined number of MD and SMD replicas. The force necessary to pull out the ligand is then used to calculate a work value (W_QB_), which corresponds to the strength of the H‐bond. What makes DUck exceptionally fast, is that only the local environment of the residue involved in the interaction is required for the simulation. Lastly, only those ligands that display the best noncovalent interactions (according to rDock and DUck) are covalently docked with CovDock using the most accurate pose prediction module.

**Tethered and constrained docking**: Using rDock, the cavity was prepared with the reference ligand method, using the respective co‐crystallized ligand. Tethered docking was used to restrain the electrophile close to the reactive residue. Tethered docking consists of two steps, namely, I) superposing atoms according to the defined SMARTS pattern, and II) docking, during which the superposed atoms can only deviate from the original position by a user‐defined cutoff. The warhead was defined by the SMARTS pattern “[#6]=[#6]−[#6]=[#8]” for the acrylamide motif. During docking, the tethered part of the ligand could move freely in terms of the dihedral degrees of freedom, while the translational and rotational degrees of freedom could deviate by max. 0.1 Å per docking run. This is meant to allow some flexibility in the sampling, also taking into consideration that the targeted cysteine residue could display a significant degree of flexibility.

Furthermore, a pose is penalized if the defined pharmacophoric constraints are not met. Here, a 1 Å deviation was permitted to increase sampling, considering that the strength of the H‐bond would still be assessed by DUck later on. For JAK3, the pharmacophoric constraints were defined as an acceptor (E903 backbone C=O) and a donor (L905 backbone NH), both of which interact with the reference ligand in 5TOZ. For KRas^G12C^, the pharmacophoric constraints were defined based on the H‐bond interactions observed between the reference ligands and the protein in the selected structures: these interactions (along with their W_QB_ values evaluated by DUck) are summarized in Table 1.

Next, the high‐throughput VS protocol (HTVS) of rDock was implemented, which consisted of three docking stages for each ligand. In each stage, the number of docking runs increases (for better sampling), and the threshold for the docking score decreases (better scores). The ligand only proceeds to the next stage if its docking score is better than the defined threshold within the specified number of runs. This is done to increase the efficiency of the simulation by progressively decreasing the number of ligands moved forward. The docking score filters were selected based on the score of the reference ligands, while being stricter for JAK3 (as the defined noncovalent interactions necessary for binding are well known) and less strict for KRas (as the defined noncovalent interactions necessary for binding are not known). For the same reason, the total number of docking runs for JAK3 was significantly lower than for KRas. The exact HTVS protocols are given in Supporting Information Schemes S2 (for JAK3) and S3 (for KRas) along with the in‐place rDock SCORE.INTER scores in Supporting Information Table S4.

**Dynamic undocking**: The first step for a DUck simulation is the definition of the chunk (a part of the protein structure) that represents the local environment surrounding the residue interacting with the ligand. Thus, for every interaction point, a separate chunk is created. When selecting residues for the chunk, the following guidelines were considered: I) selecting as little residues as possible to reduce computational time; II) residues were not selected if they would block the ligand from exiting the pocket during the simulations based on the directionality of the H‐bond; III) residues were not removed if this would lead to the possibility of solvent entering the pocket from areas other than where the ligand is exiting; and lastly IV) preserving the local environment. This was done from the already prepared structures. The gaps created during the process of selecting the chunk residues were capped. For this, each section of residues was split into separate chains, and the termini of each chain were acetylated or methylated. Lastly, the chunk was checked for clashes possibly created during the capping of the chains. The corresponding chunk definitions for JAK3 and KRas are included in Supporting Information Table S5.

After production of the chunk, DUck automatically does the following: I) automatic ligand parameterization in MOE, II) minimization, III) equilibration, and IV) a series of SMD (at two different temperatures), then MD simulations, in which the ligand is pulled from 2.5 to 5.0 Å relative to the defined H‐bond interaction. Steps II) to IV) were performed with GPU‐based pmemd.cuda in AMBER.^39^ Five replicas of step IV) were performed, during which a W_QB_ threshold of 6 kcal mol^−1^ (force necessary to pull the ligand) was maintained, so that the simulation was stopped if the measured W_QB_ value of the H‐bond was smaller. Additionally, for KRas, the inclusion of co‐crystallized GDP in the chunk was necessary, as its absence would have led to the surface being more exposed to bulk solvent. For this, GDP was parameterized using MOE′s PFROST forcefield, and the generated parameters were automatically included in the DUck protocol.

**Covalent docking with CovDock**: Because Schrödinger's CovDock pose prediction module outperformed most of the other covalent docking tools, we used this approach to rank and predict the binding mode of the virtual hits identified to have strong noncovalent interactions by the previous workflow steps.^23^ CovDock ranks the compounds according to an “Affinity Score”, which is calculated as the average of the pre‐reaction Glide score and the post‐reaction in‐place docking score. By deeming the energy of bond formation as constant across a set of compounds having the same warhead involved in the chemical reaction (as in the case of DUckCov), the affinity score can be used to compare and rank ligands in a set. CovDock affinity scores for the reference ligands of the structures that led to hits are given in Supporting Information Table S4. Contrary to the first docking step in the workflow, no additional restraints were applied in the covalent docking simulation other than those used by default in CovDock. Binding site residues were defined by centering the receptor grid on the ligand co‐crystallized in the structure under investigation. When setting up the simulation, the acrylamide warhead was automatically recognized in each ligand structure through the SMARTS‐based definition of the “Michael addition” reaction type. Ultimately, this step was incorporated to evaluate if a change in binding mode would take place upon covalent bond formation, which would prevent the ligand from establishing the interactions defined as necessary by previous workflow steps. To that end, root‐mean‐squared deviation (RMSD) values between the rDock and CovDock conformations were calculated by means of a Python script provided by Schrödinger (rmsd.py). A small RMSD, typically lower than 2.0 Å, was considered as favorable. Furthermore, the defined interaction patterns were also visually inspected for consensus.

**Biochemical and structural characterization of the identified virtual hits**: Compounds **1**, **3**, **5**, **8** and **9** were tested at 10 μm in duplicate with the Z′‐LYTE kinase inhibition assay (Life Technologies). The assay uses a fluorescence‐based format and is based on the different sensitivity of phosphorylated and non‐phosphorylated peptides to proteolytic cleavage. A suitable peptide substrate is labeled with two fluorophores (coumarin and fluorescein), forming a FRET pair. After incubating the kinase+peptide+test compound mixture for an hour, a development reaction is carried out. Any peptide that was not phosphorylated by the kinase is cleaved, disrupting the resonance energy transfer between the FRET pair. The reaction progress is quantified based on the ratio of the detected emission at 445 nm (coumarin) and 520 nm (fluorescein), that is, the ratio of cleaved versus intact peptide. A more detailed description of the assay is available on the website of Life Technologies.^40^ IC_50_ values were determined from 10 points titration measurements using the same assay.

Binding of compounds **11**–**13** and **15**–**20** to KRas^G12C^ was tested and structurally characterized by NMR measurements, performed on a Bruker Avance III 700 MHz spectrometer equipped with a 5‐mm Prodigy TCI H&F‐C/N‐D, z‐gradient probe head operating at 700.05 MHz for ^1^H and 70.94 MHz for ^15^N nuclei. ^1^H,^15^N‐HSQC spectra were recorded at 298 K to obtain the protein ^1^H and ^15^N resonances in both free and small‐molecule‐bound state and the changes in chemical shifts were followed upon complex formation. NMR samples contained ^15^N‐labeled KRas^G12C^ (catalytic domain, residues 1–169) in 150 and 50 μm concentration in free protein measurement (as a reference) and binding test, respectively, 5 mm GDP, 10 mm EDTA, 15 mm MgCl_2_ in PBS buffer, 5 % DMSO and 10 % D_2_O at pH 7.4 and 150–500 μm ligand. Because some of the ligands were not fully dissolved, we used a longer incubation time (96 h), and a high number of scans for every HSQC spectrum (NS=128). To avoid false‐positive results, the free protein was incubated for four days as well, and the spectra of the samples were compared with the spectrum of the incubated free protein. All ^1^H chemical shifts were referenced to the DMSO peaks (which were calibrated to DSS resonance before in free protein measurements) as DSS was not added to avoid any side reactions with the ligand. ^15^N chemical shift values were referenced indirectly using the corresponding gyromagnetic ratios according to the IUPAC convention. Sequence‐specific assignments of H^N^ and N in the bound KRas^G12C^ spectra were transferred from our results to be published elsewhere (BMRB entry code: 27646). There were ambiguities in a number of resonances in crowded spectral regions; however, this fact did not influence the final outcome. All spectra were processed with Bruker TOPSPIN and analyzed using NMRFAM‐SPARKY software.^41^

## Conflict of interest

*The authors declare no conflict of interest*.

## Supporting information

As a service to our authors and readers, this journal provides supporting information supplied by the authors. Such materials are peer reviewed and may be re‐organized for online delivery, but are not copy‐edited or typeset. Technical support issues arising from supporting information (other than missing files) should be addressed to the authors.

Supplementary
