# Supplementary material for: DUckCov: a Dynamic Undocking‐Based Virtual Screening Protocol for Covalent Binders
Source: ChemMedChem. 2019 Mar 8;14(10):1011–21. doi: 10.1002/cmdc.201900078 (PMC6593427; doi:10.1002/cmdc.201900078)
Supplement: Supplementary file 1 — Supplementary [file CMDC-14-1011-s001.pdf]

CHEMMEDCHEM
Chemistry & Drug Discovery

# Supporting Information

## DUckCov: a Dynamic Undocking-Based Virtual Screening Protocol for Covalent Binders

Moira Rachman,[a, b] Andrea Scarpino,[b] Dávid Bajusz,[b] Gyula Pálfy,[c] István Vida,[c] András Perczel,[c] Xavier Barril,[a, d] and György M. Keserű*[b]

cmdc_201900078_sm_miscellaneous_information.pdf

**Table of contents**

1. Switch-II loop RMSD of KRas Selected Structures ........ 2
2. rDock HTVS protocol for JAK3 ........ 2
3. rDock HTVS protocol for KRas ........ 3
4. rDock SCORE.INTER and CovDock Affinity scores for reference ligands that led to virtual hits ........ 3
5. Chunk definitions for DUck calculations ........ 4
6. 2D structures for compounds **2**, **4**, **6**, **7** and **10** ........ 5
7. JAK3 rDock+CovDock RMSD values, and CovDock, rDock and DUck scores ........ 6
8. Strain energies of the predicted binding modes of compounds **1-10** ........ 7
9. Bottom 10 rDock and CovDock poses ........ 7
10. KRas final selected compounds rDock+CovDock RMSD values, CovDock affinity and rDock SCORE.INTER scores, and DUck WQB values ........ 8
11. 2D structures for compounds **12**, **14**, **15**, **16**, **18** and **20** ........ 9
12. Strain energies of the predicted binding modes of compounds **11-20** ........ 9
13. Evidence for compounds binding to $KRas^{G12C}$ by NMR spectroscopy ........ 10
14. Comparison of the workflow with CovDock Virtual screening ........ 14

**Supplementary Table S1. Switch-II loop RMSD of KRas Selected Structures**

| PDB | 4M21 | 4M22 | 5F2E | 5V6S | 5V71 | 5V9L | 5V9O | 5V9U | 5YXZ | 5YY1 | 6ARK | 6B0V |
|---|---|---|---|---|---|---|---|---|---|---|---|---|
| **4M21** | 0 | 3.77 | 4.11 | 4.07 | 3.67 | 3.81 | 4.22 | 3.92 | 3.71 | 3.82 | 3.03 | 4.08 |
| **4M22** | 3.77 | 0 | 2.96 | 3.13 | 2.87 | 2.70 | 2.90 | 2.77 | 3.32 | 2.97 | 4.06 | 2.99 |
| **5F2E** | 4.11 | 2.96 | 0 | 0.71 | 2.23 | 2.27 | 1.32 | 1.14 | 1.96 | 0.90 | 3.49 | 0.75 |
| **5V6S** | 4.07 | 3.13 | 0.71 | 0 | 1.91 | 1.98 | 1.32 | 0.94 | 1.55 | 0.55 | 3.54 | 0.70 |
| **5V71** | 3.67 | 2.87 | 2.23 | 1.91 | 0 | 0.85 | 2.42 | 1.84 | 2.03 | 1.74 | 3.33 | 2.03 |
| **5V9L** | 3.81 | 2.70 | 2.27 | 1.98 | 0.85 | 0 | 2.34 | 1.81 | 1.95 | 1.80 | 3.64 | 1.97 |
| **5V9O** | 4.22 | 2.90 | 1.32 | 1.32 | 2.42 | 2.34 | 0 | 1.46 | 2.08 | 1.37 | 4.05 | 1.36 |
| **5V9U** | 3.92 | 2.77 | 1.14 | 0.94 | 1.84 | 1.81 | 1.46 | 0 | 1.66 | 0.65 | 3.61 | 0.69 |
| **5YXZ** | 3.71 | 3.32 | 1.96 | 1.55 | 2.03 | 1.95 | 2.08 | 1.66 | 0 | 1.50 | 3.88 | 1.63 |
| **5YY1** | 3.82 | 2.97 | 0.90 | 0.55 | 1.74 | 1.80 | 1.37 | 0.65 | 1.50 | 0 | 3.44 | 0.72 |
| **6ARK** | 3.03 | 4.06 | 3.49 | 3.54 | 3.33 | 3.64 | 4.05 | 3.61 | 3.88 | 3.44 | 0 | 3.60 |
| **6B0V** | 4.08 | 2.99 | 0.75 | 0.70 | 2.03 | 1.97 | 1.36 | 0.69 | 1.63 | 0.72 | 3.60 | 0 |

**Supplementary Scheme S2. rDock HTVS protocol for JAK3**

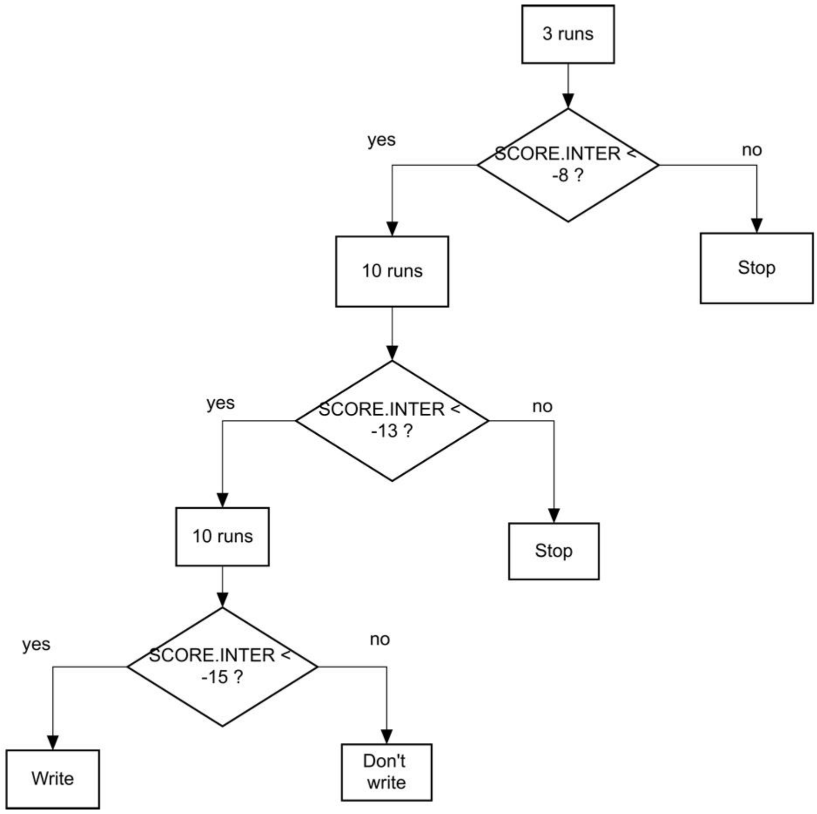

## Supplementary Scheme S3. rDock HTVS protocol for KRas

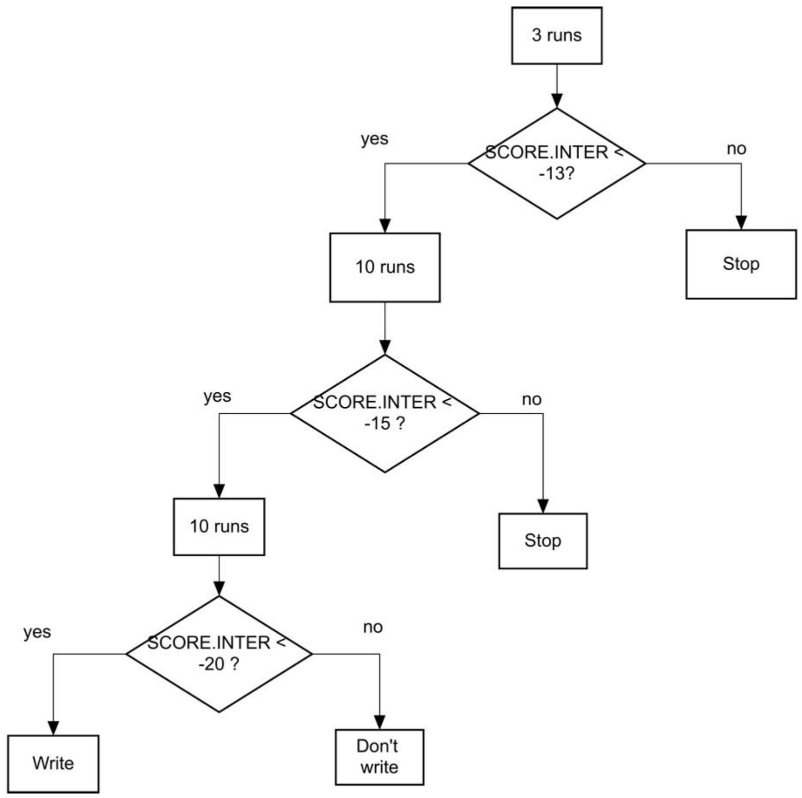


## Supplementary Table S4. rDock SCORE.INTER and CovDock Affinity scores for reference ligands that led to virtual hits

| PDB | LIG | Chain | rDock SCORE.INTER | CovDock Affinity Score |
|---|---|---|---|---|
| **JAK3** | | | | |
| 5TOZ | 7H4 | A | -12 | -8.7 |
| **KRas** | | | | |
| 5V6S | 8YD | A | -32 | -11 |
| 5V9O | 91G | A | -30 | -9.8 |

**Supplementary Table S5. Chunk definitions for DUck calculations**

| PDB ID | Pull residue | Pull atom | Kept residues |
|---|---|---|---|
| **JAK3** | | | |
| 5TOZ | E903 | O | 826-831, 834-838, 852-855, 871, 875, 884-885, 900, 902-913, 951-957, 966-969 |
| 5TOZ | L905 | N | 826-831, 834-838, 852-855, 871, 875, 884-885, 900, 902-913, 951-957, 966-969 |
| **KRas$^{G12C}$** | | | |
| 4M22 | K16 | NZ | 6-40, 55-61, 71, 78-96, 100, 112-120, 142-159,GDP |
| 5F2E | R68 | NH2 | 5-7, 37, 56-78, 99-104 |
| 5F2E | D69 | OD1 | 62-78, 95-106 |
| 5F2E | E63 | O | 12- 34-37, 56-72, 96-103 |
| 5F2E | K16 | NZ | 6-40, 55-60, 68, 72, 78-96, 100, 112-120, 141-159,GDP |
| 5V6S | K16 | NZ | 7-21, 29-40, 55-60, 68, 72, 79-94, 96, 114-117, 146, 156,GDP |
| 5V6S | D69 | OD1 | 62-74, 78, 98-105 |
| 5V6S | H95 | NE2 | 9-14, 62-64, 80-82, 87-102, 111-113, 133, 137 |
| 5V71 | H95 | NE2 | 8-16, 58, 72, 78-103, 107-115, 125, 129-141 |
| 5V71 | K16 | NZ | 7-23, 28-40, 55-60, 68, 79-89, 93-96, 114- 117, 144-147, 152, 156,GDP |
| 5V9L | K16 | NZ | 6-41,54-62,78-90,92-97,113-118,143,144,145-148,151-153,155-157,GDP |
| 5V9L | H95 | NE2 | 8-15,79-83,86-103,110-114,132-134,136-140 |
| 5V9O | K16 | NZ | 6-23,27-41,54-63,67-69,71-73,77-97,99-118,143-147,151-153,155-157,GDP |
| 5V9O | E63 | OE | 6-17,57,58-105,108-115,124-126,129,130-134-140 |
| 5V9O | D69 | OD1 | 59-79,96-106 |
| 5V9U | H95 | NE2 | 8-17,61-63,77,78-103,110-117,124-126,128-142 |
| 5YXZ | K16 | NZ | 7-21,56-59,80-84,115-118,GDP |
| 5YXZ | H95 | NE2 | 8-17,61-63,71-73,77,78,79,80-104,110-115,124-126,128,129-140 |
| 5YY1 | K16 | NZ | 7-21,56,57-59,80,81-84,115-118,145-147, GDP |
| 5YY1 | H95 | NE2 | 8-15,61,62-65,79,80-83,86-103,110-114,132-134,136-138 |
| 6B0V | R68 | NH2 | 6-8,36-38,55-62,67-73 |
| 6B0V | K16 | NZ | 7-23,27-37,55-60,78-90,92-97,113-118,143,144-147,151-153,155-157,GDP |

**Supplementary Figure S6.** 2D structures for compounds **2**, **4**, **6**, **7** and **10** (compounds from case study 1 not tested).

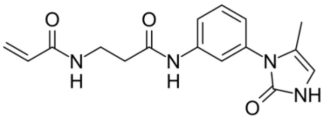

**2**

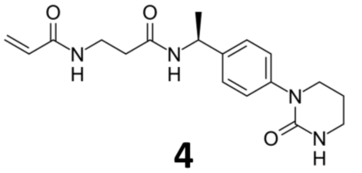

**4**

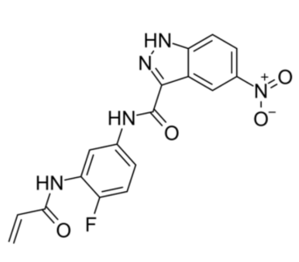

**6**

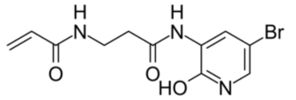

**7**

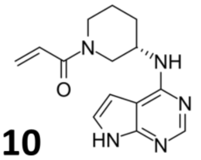

**10**

**Supplementary Table S7. JAK3 RMSD values, and CovDock, rDock and DUck scores**

| ID | RMSD rDock+ CovDock Pose | CovDock Affinity Score | rDock SCORE.INTER | DUck E903 WQB value | DUck L905 WQB value |
|---|---|---|---|---|---|
| **Top 10** | | | | | |
| **1** (ZINC000204947687) | 2.1 | -9.9 | -23 | 16 | 12 |
| **2** (ZINC000161074700) | 5.3 | -9.1 | -17 | 9.3 | 11 |
| **3** (ZINC000171805058) | 4.8 | -9.1 | -15 | 11 | 9.2 |
| **4** (ZINC000292089827) | 2.5 | -9.0 | -15 | 6.2 | 7.5 |
| **5** (ZINC000136550518) | 0.8 | -8.8 | -22 | 12 | 11 |
| **6** (ZINC000176723207) | 3.6 | -8.6 | -22 | 13 | 11 |
| **7** (ZINC000490601242) | 0.94 | -8.5 | -15 | 10 | 8.8 |
| **8** (ZINC000490676647) | 1.5 | -8.5 | -15 | 12 | 12 |
| **9** (ZINC000490581921) | 0.73 | -8.5 | -21 | 8.6 | 11 |
| **10** (ZINC000584627637) | 2.6 | -8.5 | -18 | 9.8 | 15 |
| **Bottom 10** | | | | | |
| 1. ZINC000156427906 | 4.4 | -6.6 | -16 | 7.6 | 11 |
| 2. ZINC000490704970 | 5.1 | -6.5 | -22 | 10 | 14 |
| 3. ZINC000584619265 | 6.3 | -6.4 | -17 | 8.3 | 11 |
| 4. ZINC000080172744 | 4.9 | -6.4 | -17 | 6.2 | 6.3 |
| 5. ZINC000490760387 | 4.4 | -6.0 | -16 | 8.2 | 9.3 |
| 6. ZINC000490584835 | 8.0 | -5.9 | -19 | 6.1 | 7.2 |
| 7. ZINC000490673765 | 1.9 | -5.6 | -18 | 8.9 | 7.7 |
| 8. ZINC000136562025 | 7.4 | -5.6 | -15 | 7.7 | 6.8 |
| 9. ZINC000490676038 | 1.5 | -5.5 | -19 | 11 | 7.0 |
| 10. ZINC000490678217 | 8.9 | -4.7 | -16 | 6.3 | 8.1 |

*ranked according to CovDock affinity score

**Supplementary Table S8.** Strain energies of the predicted binding modes of compounds **1-10** (compared to minimum energy conformations of the free ligands).

| Compound # | Strain Energy [kcal/mol] of CovDock pose | Strain Energy [kcal/mol] of rDock pose | Δ(Strain Energy) | RMSD [rDock vs CovDock] |
|---|---|---|---|---|
| 1 | 12.67 | 14.02 | 1.35 | 2.11 |
| 2 | 6.26 | 10.89 | 4.63 | 5.29 |
| 3 | 4.14 | 4.57 | 0.43 | 4.84 |
| 4 | 9.53 | 13.72 | 4.19 | 2.45 |
| 5 | 6.42 | 2.04 | -4.38 | 0.8 |
| 6 | 16.04 | 1.96 | -14.08 | 3.6 |
| 7 | 0.69 | 4.59 | 3.9 | 0.94 |
| 8 | 7.07 | 2.69 | -4.38 | 1.53 |
| 9 | 5.47 | 2.18 | -3.29 | 0.73 |
| 10 | 10.92 | 5.73 | -5.19 | 2.62 |

**Supplementary Figure S9. Bottom 10 rDock and CovDock poses**

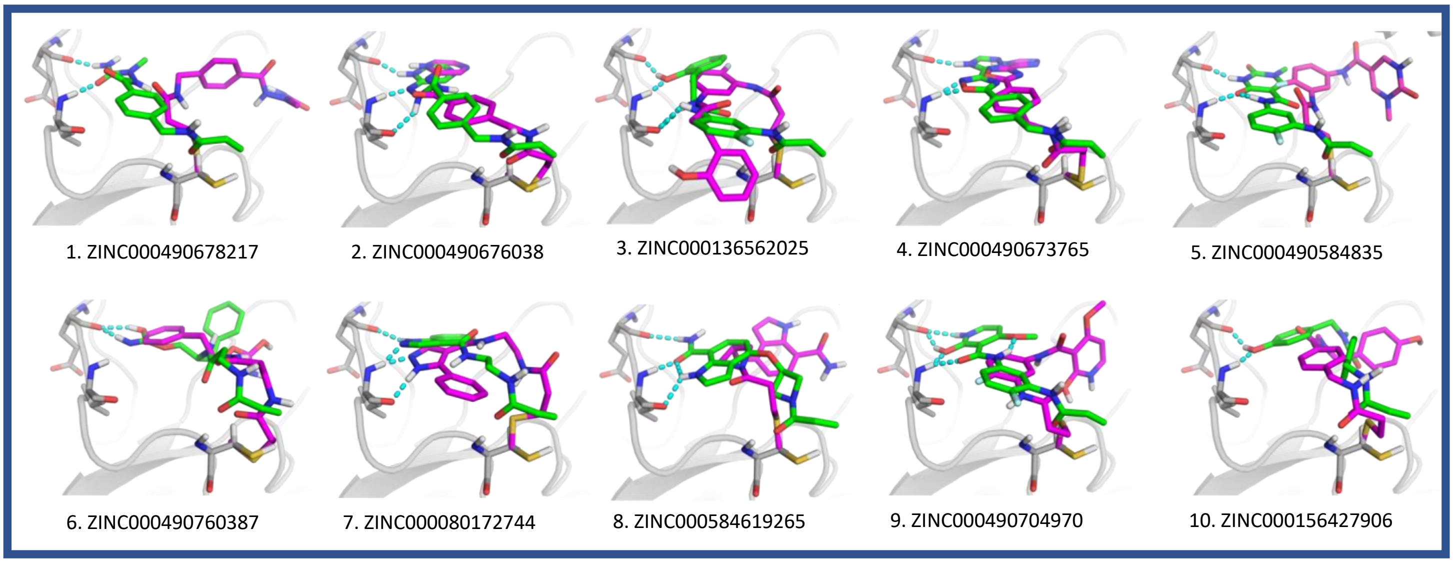

**Supplementary Table S10. KRas final selected compounds rDock+CovDock RMSD values, CovDock affinity and rDock SCORE.INTER scores, and DUck WQB values**

| ID | RMSD rDock+ CovDock Pose | CovDock Affinity Score | rDock SCORE.INTER | DUck WQB value |
|---|---|---|---|---|
| **5V6S D69-OD1** | | | | |
| **11** (ZINC00058823329) | 1.4 | -7.7 | -20 | 7.2 |
| **12** (ZINC000157292437) | 2.1 | -8.3 | -22 | 12 |
| **13** (ZINC000136603796) | 2.7 | -7.0 | -22 | 14 |
| **14** (ZINC000490670179) | 1.7 | -7.6 | -22 | 7.8 |
| **15** (ZINC000490557519) | 1.0 | -7.0 | -20 | 7.5 |
| **16** (ZINC000264777750) | 2.3 | -8.4 | -20 | 12 |
| **5V9O E63-OE2** | | | | |
| **17** (ZINC000366691596) | 1.1 | -5.8 | -23 | 10 |
| **5V9O D69-OD1** | | | | |
| **18** (ZINC000136617934) | 5.1 | -6.6 | -25 | 9.8 |
| **19** (ZINC000162217082) | 1.6 | -7.0 | -25 | 10 |
| **20** (ZINC000156629354) | 1.7 | -6.2 | -26 | 7.5 |

**Supplementary Figure S11.** 2D structures for compounds **2**, **4**, **6**, **7** and **10** (compounds from case study 2 not tested or not confirmed as experimental hits).

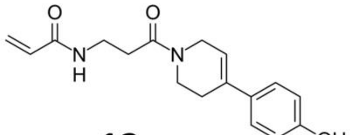

**12**

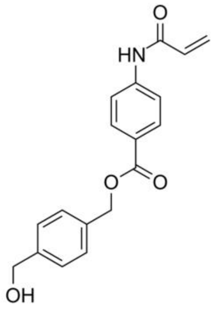

**14**

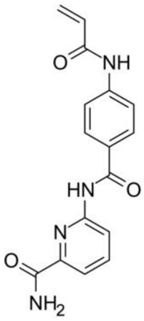

**15**

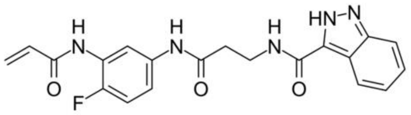

**18**

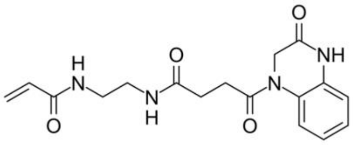

**16**

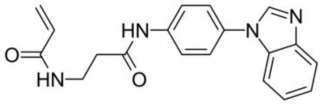

**20**

**Supplementary Table S12.** Strain energies of the predicted binding modes of compounds **11-20** (compared to minimum energy conformations of the free ligands).

| Compound # | Strain Energy [kcal/mol] of CovDock pose | Strain Energy [kcal/mol] of rDock pose | Δ(Strain Energy) | RMSD [rDock vs CovDock] |
|---|---|---|---|---|
| 11 | 2.15 | 36.67 | 34.52 | 1.42 |
| 12 | 4.07 | 21.74 | 17.67 | 2.07 |
| 13 | 4.04 | 20.08 | 16.04 | 2.67 |
| 14 | 2.16 | 0.57 | -1.59 | 1.72 |
| 15 | 5.83 | 4.8 | -1.03 | 1.05 |
| 16 | 6.7 | 17.91 | 11.21 | 2.33 |
| 17 | 10.73 | 6.58 | -4.15 | 1.1 |
| 18 | 31.75 | 4.19 | -27.56 | 5.1 |
| 19 | 6.32 | 8.16 | 1.84 | 1.63 |
| 20 | 2.94 | 2.53 | -0.41 | 1.7 |

**Supplementary Figure S13.** Full $^{1}H,^{15}N$-HSQC spectra of compounds **11-13** and **15-20** with $KRas^{G12C}$ in GDP-bound form, after incubation, measured at 700 MHz at 298 K. (Red: reference spectra of free $KRas^{G12C}$, blue: $KRas^{G12C}$ + ligand).

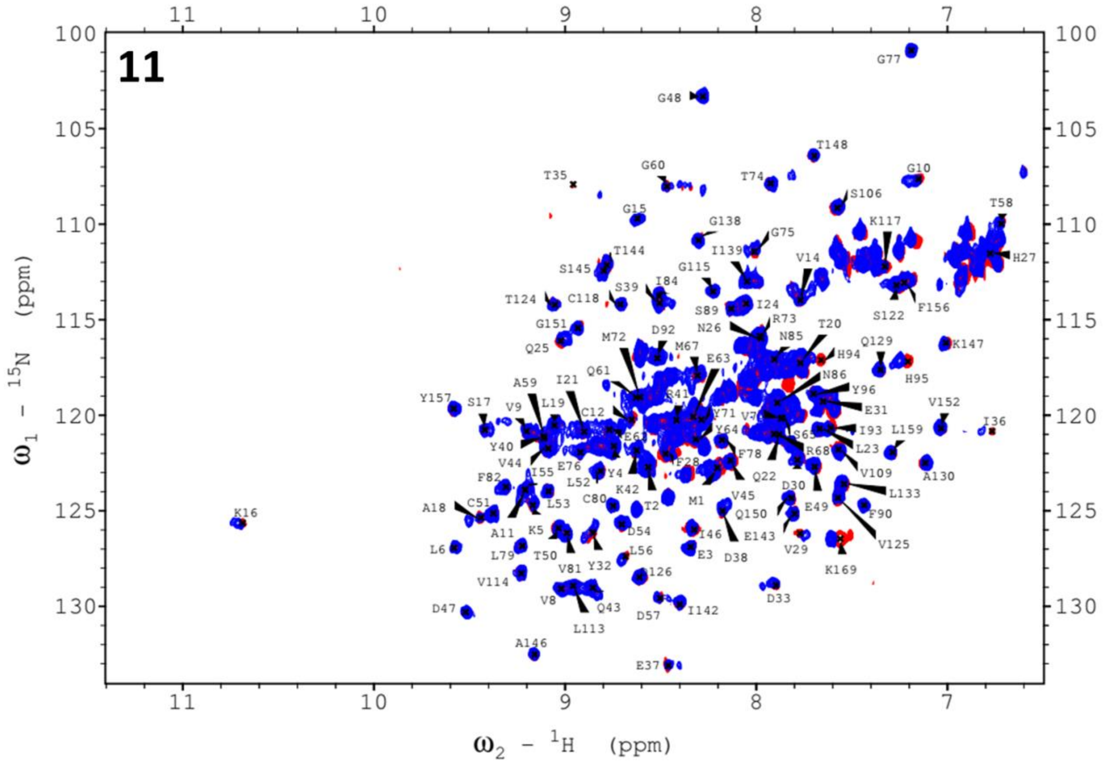


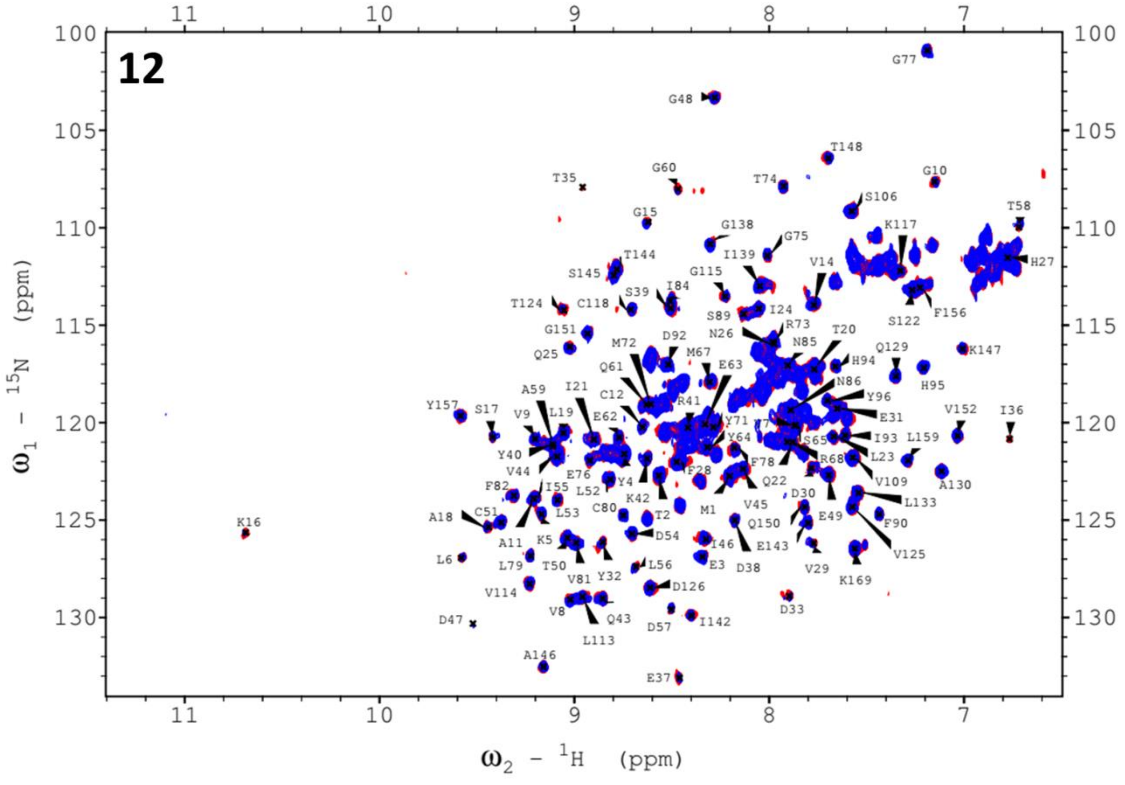

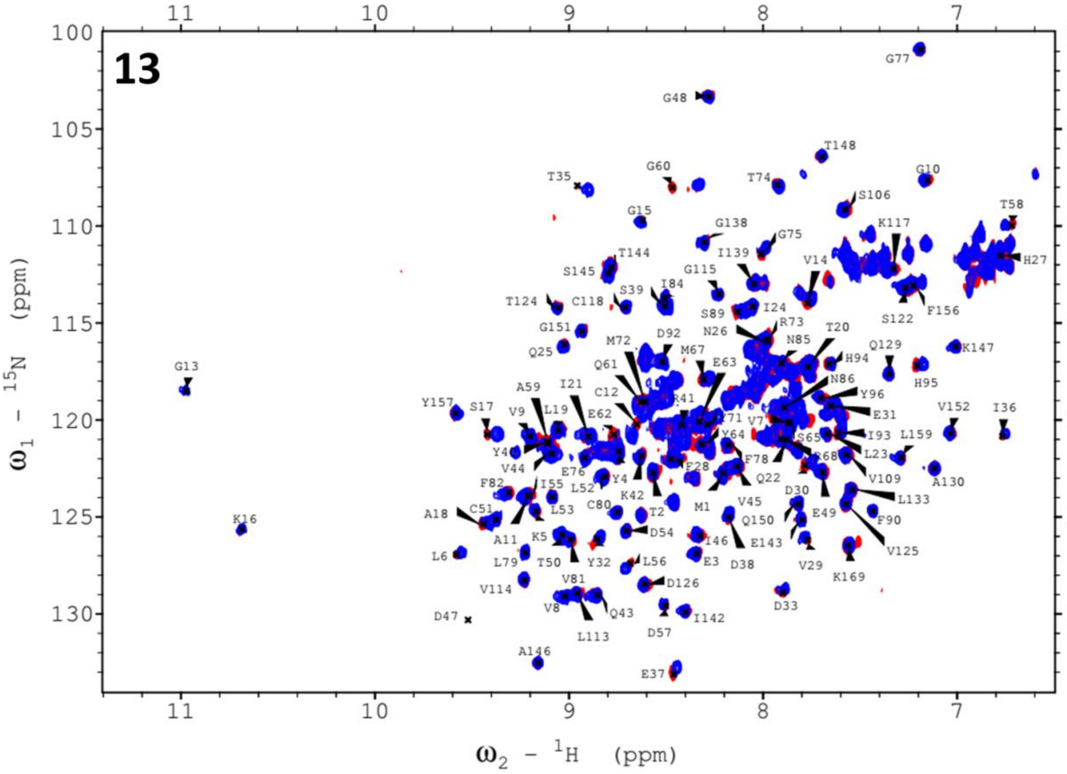
13
ω₂ - ¹H (ppm)
ω₁ - ¹⁵N (ppm)

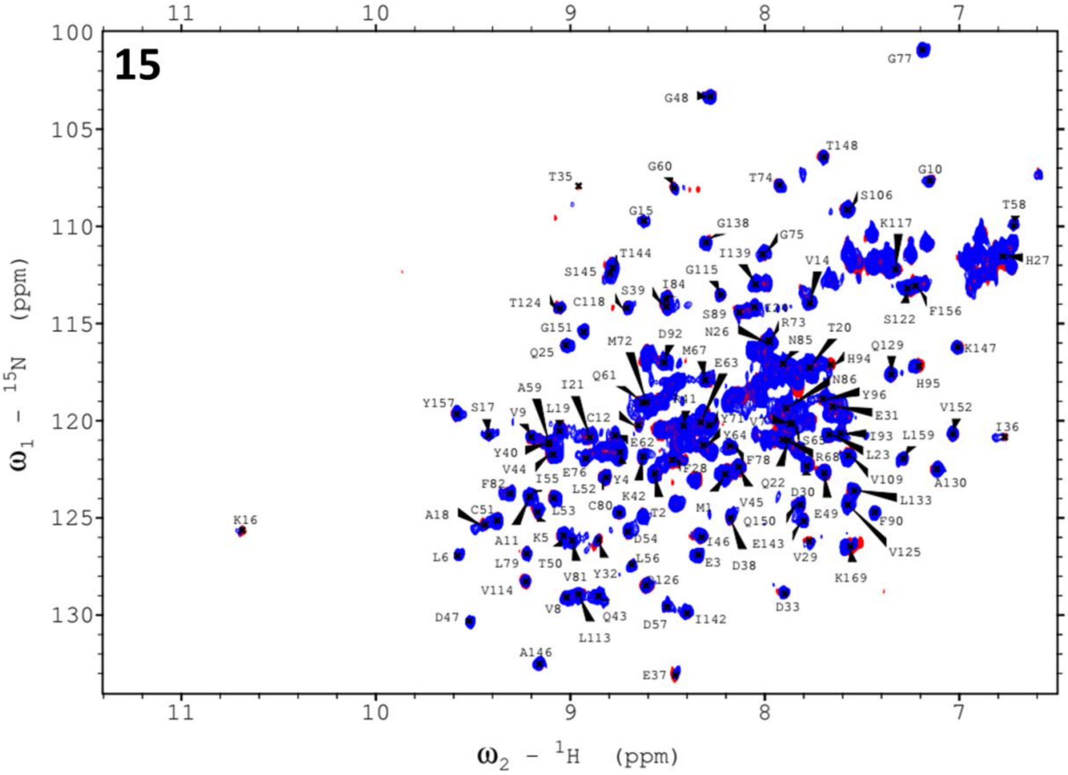
15
ω₂ - ¹H (ppm)
ω₁ - ¹⁵N (ppm)

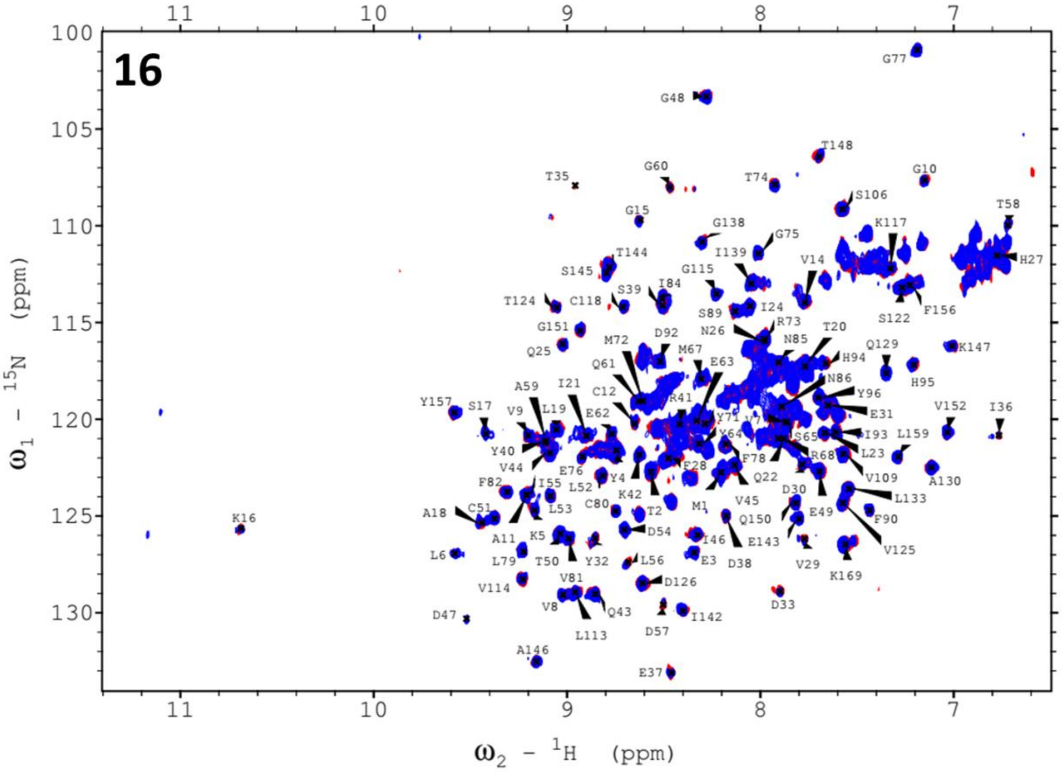
16
ω1 - 15N (ppm)
ω2 - 1H (ppm)

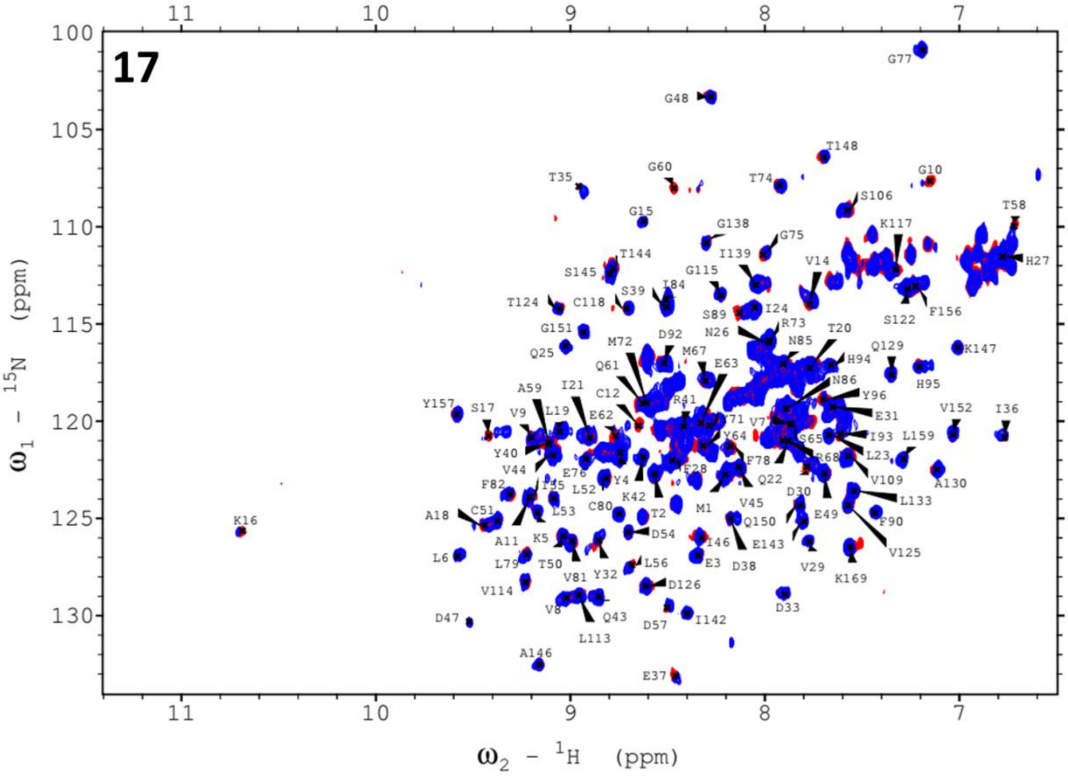
17
ω1 - 15N (ppm)
ω2 - 1H (ppm)

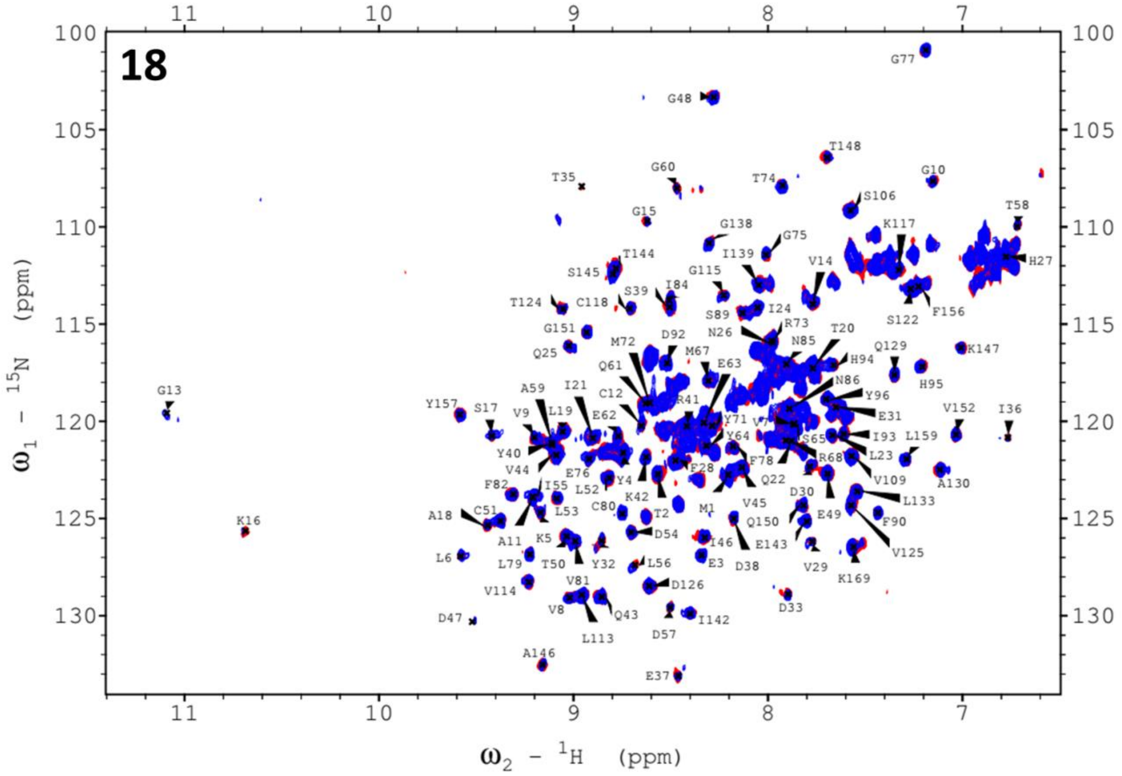
18
ω1 - 15N (ppm)
ω2 - 1H (ppm)

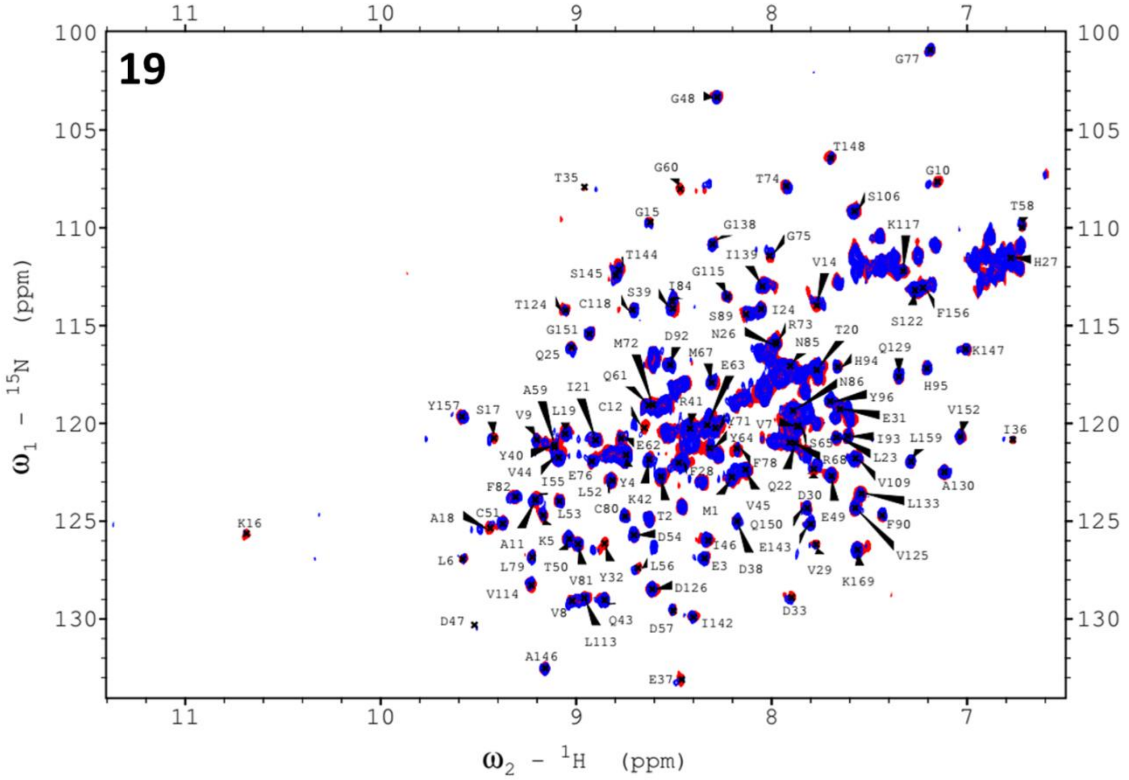
19
ω1 - 15N (ppm)
ω2 - 1H (ppm)

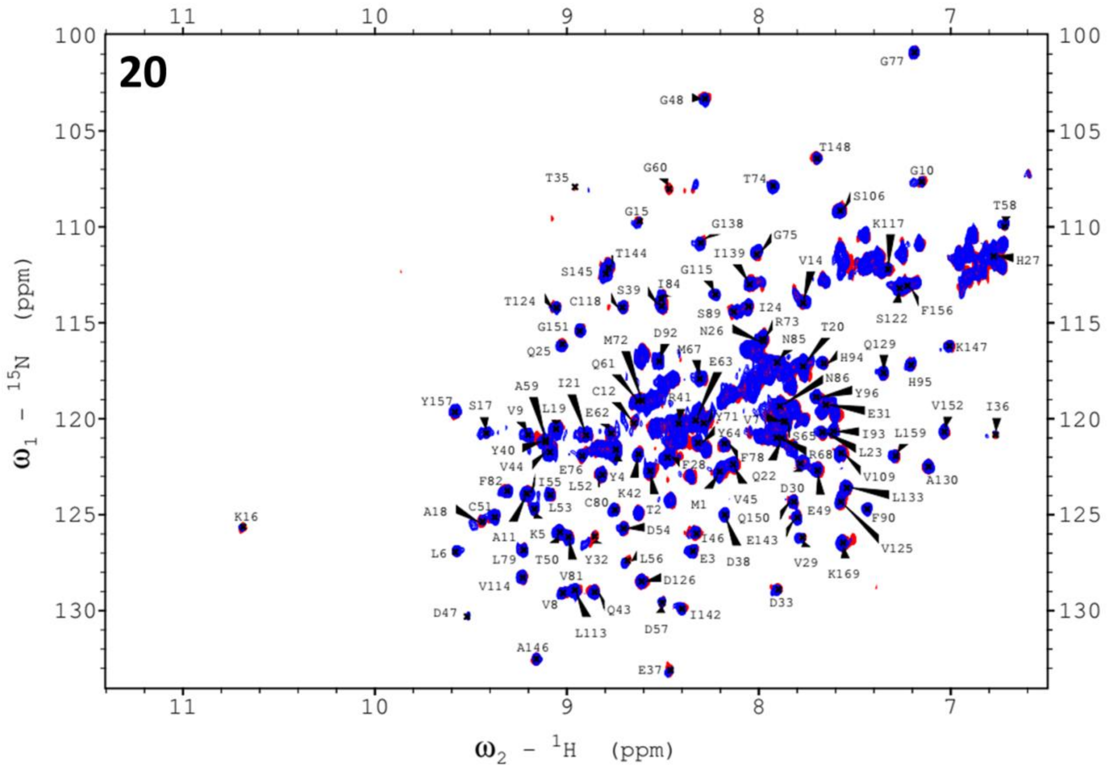


**Supplementary Table S14.** Comparison of the workflow with CovDock Virtual screening

| Compound # | Ranking in CovDock VS | Target | Structure |
|---|---|---|---|
| 1 | 1 | JAK3 | 5TOZ |
| 5 | 8 | JAK3 | 5TOZ |
| 9 | 14 | JAK3 | 5TOZ |
| 11 | 9 | KRAS | 5V6S |
| 13 | 34 | KRAS | 5V6S |
| 17 | 337 | KRAS | 5V9O |
| 19 | 109 | KRAS | 5V9O |

As a comparison, we used CovDock-VS to screen the hits identified by tethered docking with rDock (the first step of the workflow) against JAK3 and KRAS. In this table, we provide the ranking obtained with CovDock-VS for the actives retrieved by DUckCov (and reported in the manuscript).

Two out of the three JAK3 actives (1 and 5) were found also by CovDock-VS among the top 10 scoring ligands. This is in line with the expected outcome, as the binding mode

adopted by kinase inhibitors is characterized by well-known interaction patterns at the hinge region. Consequently, highly similar binding modes can be predicted by both modules in CovDock ("pose prediction" and "virtual screening"), thus also providing similar rankings.

On the other hand, the improvement guaranteed by DUckCov is clear in the case of a more challenging target like KRAS$^{G12C}$. The covalent binders identified by our protocol were not ranked among the top scoring solutions by CovDock-VS. This highlights the importance of DUck in the prioritization of compounds predicted to form strong hydrogen bond interactions in the pocket, prior to covalent docking with algorithms providing more accurate binding mode predictions.
